# Proteome profiling of lipopolysaccharide induced L6 rat skeletal muscle cells response to flavonoids from *Scutellaria baicalensis* Georgi

**DOI:** 10.1186/1472-6882-14-379

**Published:** 2014-10-07

**Authors:** Jin A Kim, Arulkumar Nagappan, Hyeon Soo Park, Venu Venkatarame Gowda Saralamma, Gyeong Eun Hong, Silvia Yumnam, Ho Jeong Lee, Suchismita Raha, Eun Hee Kim, Paik Soon Young, Gon Sup Kim

**Affiliations:** Department of Microbiology, College of Medicine, The Catholic University of Korea, Seoul, 137-701 Republic of Korea; Research Institute of Life Science and College of Veterinary Medicine (BK21 plus project), Gyeongsang National University, Gazwa, Jinju, 660-701 Republic of Korea; Department of Nursing Science, International University of Korea, Jinju, 660-759 Republic of Korea

**Keywords:** Flavonoids, *Scutellaria baicalensis* G, L6 skeletal muscle cells, Lipopolysaccharide (LPS), Two dimensional gel electrophoresis, Matrix assisted laser desorption ionization time of flight mass spectrometry (MALDI-TOF/MS)

## Abstract

**Background:**

*Scutellaria baicalensis* Georgi is a commonly used medicinal herb in several Asian countries like Korea, China and Japan for thousands of years. It has been reported to have various medicinal properties such as anti-microbial, anti-inflammatory and anti-cancer effects. However, the anti-inflammatory mechanism of *S. baicalensis* G at proteome level has not yet been reported. Hence, we performed a proteome analysis to study differentially expressed proteins and its anti-inflammatory role in lipopolysaccharide (LPS) stimulated L6 skeletal muscle cells response to flavonoids isolated from *S. baicalensis* G.

**Methods:**

For that, 150 μg of proteins from the L6 cells of the control (Vehicle only), LPS treated and flavonoid treated groups were separated using 18 cm, pH 4–7 IPG strips in the first dimension and resolved by 12% linear gradient SDS-polyacrylamide gel electrophoresis (SDS-PAGE). The silver stained gels were analyzed by using progenesis SameSpots software and twenty six differentially expressed protein spots (≥2 fold, p < 0.05) were selected for matrix assisted laser desorption ionization- time of flight mass spectroscopy/mass spectrometry (MALDI-TOF/MS) analysis. Also, the expression of COX-2, iNOS and Annexin A2 proteins were analyzed by western blot.

**Results:**

Totally, 12 differentially expressed proteins were successfully identified by MALDI-TOF/MS and database searching, that’s involved in inflammatory responses such vimentin, T-box transcription factor TBX3, annexin A1, annexin A2 and annexin A5. In addition, flavonoids inhibited the expression of COX-2, iNOS and Annexin A2 proteins in LPS-stimulated L6 skeletal muscle cells.

**Conclusions:**

The findings revealed that the flavonoids from *S. baicalensis* G. directly protect the LPS stimulated inflammation process in L6 cells and, would be helpful to study further the muscle cell inflammatory mechanism. This is the first proteome study provide the anti-inflammatory mechanism of flavonoids from *S. baicalensis* G. in LPS stimulated L6 skeletal muscle cells.

## Background

*Scutellaria baicalensis* Georgi (Lamiaceae) is one of the most widely used herbs in Traditional Chinese Medicine (TCM) for thousands of years. It’s possesses various biological activities such as anti-microbial, anti-inflammatory and anti-cancer effects [1, 2]. The bioactive compounds present in *S. baicalensis* are the flavones like baicalin (5,6-dihydroxy-4-oxygen-2-phenyl- 4H-1-benzopyran-7-beta-d-glucopyranose acid), baicalein (5,6,7-trihydroxy-2-phenyl-4H-1-benzopyran-4-one), scutellarin and wogonin (5,7-dihydroxy-8- methoxyflavone) and [3–5]. Wogonin, the main active component which exhibits anti-cancer activities [6, 7], anti-angiogenesis [8], and also has been reported to inhibit cell growth and induces apoptosis in various cancer cell lines [9–11].

In addition, Baicalein, other major flavonoids in *S. baicalensis* G., widely used to treat cancer and various inflammatory diseases [12, 13]. Baicalin inhibits various human cancer cell growth in-vivo [7, 14, 15]. Recent studies have demonstrated that the role of *S. baicalensis* G. in cancer cell line, including cell cycle arrest and apoptosis [16, 17]. Our recent studies have shown that the water extract and flavonoids from Korean *S. baicalensis* G. inhibits cell cycle G1/S transition in A549 lung cancer cells [18] and inhibited inflammatory signaling pathways in RAW 264.7 Cells, respectively [19]. However, anti-inflammatory effect of Korean *S. baicalensis* G. in LPS induced L6 skeletal muscle cells using proteomic approach has not been studied. Hence, in the present study, we performed a proteome analysis of L6 cells after treatment with LPS and flavonoids Korean *S. baicalensis* G. and 12 differentially expressed proteins were identified by MALDI-TOF/MS. These proteome results suggest that flavonoids from *S. baicalensis* G. directly protect the LPS stimulated inflammation process in L6 skeletal muscle cells. To our knowledge, this is the first study provide the evidence for an interaction between flavonoids from *S. baicalensis* G. and LPS stimulated L6 skeletal muscle cells.

## Methods

### Chemicals and reagents

Dulbecco’s modified Eagle’s medium was purchased from Hyclone (Logan, UT, USA). Fetal bovine serum (FBS) and antibiotics (penicillin/streptomycin; P-S) were purchased from Gibco (BRL Life Technologies, Grand Island, NY, USA). Materials and chemicals used for electrophoresis were obtained from BioRad (Hercules, CA, USA). Antibodies to COX-2 and iNOS were obtained from Santa Cruz Biotechnology (Santa Cruz, CA, USA). Annexin A2 was purchased from Cell Signaling Technologies (Beverly, MA, USA) and ß-actin was purchased from Millipore (Billerica, MA, USA). All other chemicals were purchased from AMRESCO (Solon, OH, USA) and Sigma-Aldrich (St. Louis, MO, USA). All the chemicals used were of the highest grade available commercially.

### Preparation of SBWE and isolation of flavonoids

*Scutellaria baicalensis* cultivated in Korea was obtained from the Animal Bio-Resources Bank (Jinju, Korea). The voucher specimen (#00100B) was deposited at the Animal Bio Resources Bank, Gyeongsang National University. The flavonoids and plant material used in this study were supported by the Department of Chemistry, Gyeongsang National University by Prof. Sung Chul Shin. High performance liquid chromatography (HPLC) was performed as described previously [9]. The mass spectrometer was operated in the positive mode with selected ion monitoring using BioAnalyst™, version 1.4.2 (AB Sciex, Zagreb, Croatia). Electron spray voltage was set at 5.5 kV, and the source temperature was 400°C. Mass spectra were recorded between m/z 100 and m/z 1500 with a step size of 0.1 amu. Samples were stored at -20°C until used for various cell culture treatments.

### Cell culture and evaluation of cell viability by MTT assay

L6 Rat Skeletal Muscle Cells were obtained from Korean Cell Line Bank (KCLB, Seoul, Korea) and cultured in DMEM medium, supplemented with 10% FBS and 1% P-S, and grown in a humidified incubator with 5% CO_2_ in air at 37°C. The L6 cells were cultured in 12-well plates and incubated overnight. The cells were pretreated with 30, 60, 90, 120 and 150 *μ*g/mL flavonoids for 1 h and then treated with LPS (1 *μ*g/mL) for 24 h. The cells were incubated in 100 *μ*L MTT solution (5 mg/mL in phosphate buffered saline; PBS) at 37°C for 3 h. The violet crystal deposits were dissolved with 500 *μ*L dimethyl sulfoxide and absorbance was read at 540 nm using an enzyme-linked immunosorbent assay (ELISA) microplate reader. All experiments were conducted in triplicate.

### Sample preparation for 2D-polyacrylamide gel electrophoresis (2-DE)

Whole protein extracts were prepared from control, LPS treated, and flavonoids treated L6 cells. Briefly, cell pellets were resuspended in 500 μl of lysis buffer (7 M urea, 2 M thiourea and 4% w/v CHAPS) on ice for 1 h. The lysates were centrifuged at 15000 rpm for 15 min, collected the supernatant and stored at -80°C until analysis. The proteins in the supernatant was precipitated with 10% TCA (v/v) and incubated for 1 h at -20°C. The samples were then washed with ice cold acetone and protein pellets were dried in a lyophilizer dryer (SFDSM06, Samwon Freezing Engineering Co., Busan), dissolved in 500 μl of sample buffer and stored at 80°C until further analysis. Protein concentration was determined by the NI™ (Non-Interfering™) Protein Assay kit (G-Biosciences, St Louis, MO, USA) according to the manufacturer’s protocol.

### 2-DE and image analysis

The 150 μg of proteins from all three groups were rehydrated overnight using 18 cm IPG strips (pH 4–7; Amersham Biosciences) at room temperature in the first dimension. The protein samples were focused with a Pharmacia Multiphor II separation unit (Amersham Biosciences) for 67.2 kVh. The focused strips were equilibrated twice for 15 min each time, first in 10 mg/ml dithiothreitol (DTT) and then in 40 mg/ml iodoacetamide (IAA) prepared in an equilibration buffer containing 50 mM Tris–HCl (pH 8.8), 6 M urea, 30% (v/v) glycerol, and 2% (w/v) sodium dodecyl sulfate (SDS). The focused proteins were then separated in the second dimension by 12% linear gradient SDS-polyacrylamide gel electrophoresis (SDS-PAGE) with a constant current of 25 mA/gel at 20°C. Gels were run until the bromophenol dye front reached the bottom of the gel. The silver stained gels were scanned using Bio Rad GS-800 densitometer and analyzed by using progenesis SameSpotsTM 2D software (ver. 4.1, Nonlinear Dynamics, Newcastle, U.K.). Protein spots showing ≥ 2 fold and p < 0:05 changes in expression were considered as statistically significant altered proteins.

### In-gel protein digestion, MALDI-TOF-MS analysis and database searching

The statistically significant protein spots were excised manually from each 2-DE gel and the protein was digested as previously described by [20] with slight modifications. The excised protein spots were proteolyzed in-gel with trypsin and resulting tryptic peptides were subsequently extracted and dried using a vacuum freeze dryer (SFDSM06, Samwon Freezing Engineering Co., Busan). Finally, the extracts were targeted onto a MALDI plate and MALDI-TOF MS were performed on a Voyager- DE STR mass spectrometer (Applied Biosystems) equipped with delayed ion extraction. Mass spectra were obtained over a mass range of 800–3,500 Da. For identification of proteins, the peptide mass fingerprinting data were used to search against NCBI non-redundant protein database using the Mascot program (http://www.matrixscience.com). The following parameters were used for database searches: taxonomy, *Rattus*; cleavage specificity, trypsin with 1 missed cleavages allowed; peptide tolerance of 100 ppm for the fragment ions; allowed modifications, Cys Carbamidomethyl (fixed), oxidation of Met (variable). The proteins were identified as significant hits (P < 0.05) by a peptide mass fingerprinting (PMF) ion search. To identify the correct protein from a Mascot results list, MOWSE score and species had to be considered.

### Western blot analysis

The L6 cells were cultured in wells of 6-well plates were incubated in DMEM as the solvent control or, LPS treated, and flavonoids (150 *μ*g/mL) for 24 h at 37°C. After washing with cold PBS, the cells were lysed in RIPA buffer [1% (w/w) NP40, 1% (w/v) sodium deoxycholate, 0.1% (w/v) SDS, 0.15 M NaCl, 0.01 M sodium phosphate buffer, pH 7.2, 2 mM EDTA, and 50 mM Naf (as phosphatase ingibitor) and protease inhibitors]. The cell debris was removed by centrifugation at 14,000 rpm for 25 min and protein concentration was determined using a Bradford assay (Bio-Rad). Proteins were separated by 12% SDS-polyacrylamide gel electrophoresis (SDS-PAGE) and transferred to a polyvinyldene fluoride (PVDF) membrane (Immunobilon-P, 0.45 mm; Millipore, Billerica, MA, USA) using the TE 77 Semi-Dry Transfer Unit (GE Healthcare Life Sciences, Buckinghamshire, UK). The membranes were blocked with 5% non-fat skim milk in Tris-buffered saline containing 0.1% Tween 20 (TBS-T, pH 7.4) at room temperature for 1 h. Then, each membrane was incubated with primary antibody (1:1000 dilution) for overnight at 4°C, washed 5 times for 10 min each time with TBS-T, and incubated with HRP-conjugated secondary antibody (1:2000 dilution) for 3 h at room temperature. Each membrane was washed 5 times for 10 min each time with TBS-T. The membranes were developed using an enhanced chemiluminescence (ECL) kit (GE Healthcare Life Sciences, Buckinghamshire, UK).

### Gene ontology (GO) analysis

The Spot identities were submitted to GORetriever (http://www.agbase.msstate.edu/) to obtain the GO annotations. If no annotation was returned, GOanna was used to retrieve GO annotations assigned depending on the sequence similarities. The resulting annotations were summarized based on the GOA and whole proteome GOSlim set using GOSlimViewer [21]. These obtained results were exported to an Excel file and percentile calculations were done. The data is presented pie chart form to ease understanding.

### Statistical analysis

The obtained results were expressed as the mean ± standard deviation. Differences between the control, LPS and flavonoids treated groups were determined using one-way analysis of variance followed by a Student’s t-test with p < 0.05 as the limit of significance. All statistical analyses were performed using SPSS software (SPSS for Windows, ver. 10.0; SPSS Inc. Chicago, IL, USA).

## Results

### Quantification and characterization of flavonoids from *Scutellaria baicalensis*G

**Figure 1:**
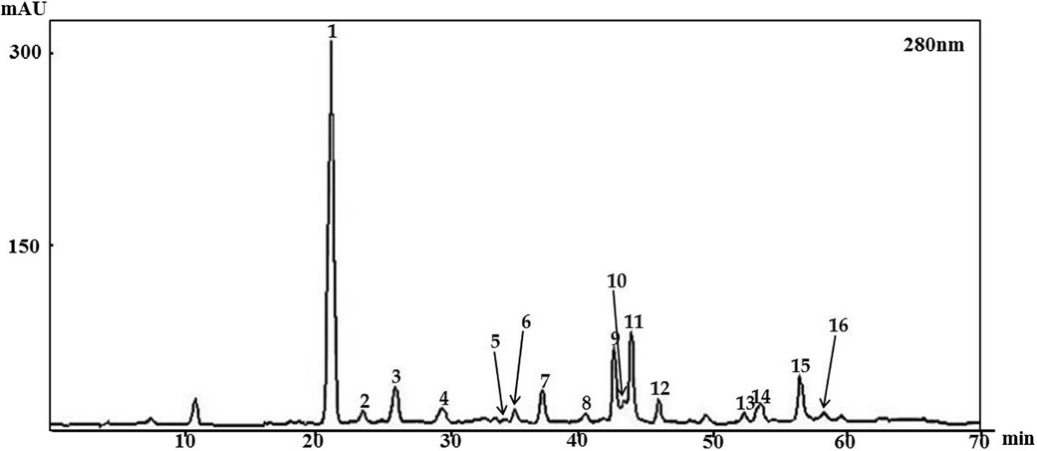
**High performance liquid chromatography chromatogram.** Totally 16 flavonoid compounds identified from Korean *S. baicalensis* G. at 280 nm and that’s listed in Table 1.

Flavonoids were isolated from Korean *S. bicalensis* G. using HPLC-MS/MS. totally 16 peaks were identified based on the HPLC retention time, molecular ion masses and the ultraviolet–visible spectra of standard compounds in a library. It was confirmed according to the peaks of HPLC chromatogram, that flavonoid components were in the extract at 280 nm (Figure 1). These flavonoid compounds are the essential components present in Korean *S. baicalensis* G. The mass-spectral and quantification data are presented in Table 1.

**Table 1:** **Spectral data of the 16 flavonoids identified from Korean** ***S. baicalensis*** **G. using HPLC-MS/MS**

No	Compound	Rt (min)	[M-H] ^-^/[M + H] ^+^	MS/MS	^a^Quantity (mg/kg)
1	Pentahydroxyflavanone derivative	20.64	629	303 285 275 217 201 177 149 125	212.543 ± 1.074
2	Pentahydroxyflavanone	23.49	303	303 285 275 259 217 193 177 149 125 109	16.670 ± 0.188
3	Viscidulin I –*O*– diglucoside	25.98	625	301 283 273 258 229 185 151 125	21.220 ± 0.440
4.	Pentahydroxyflavone	29.31	301	301 283 269 259 240 191 179 161 139 124 121 109	17.657 ± 0.204
5	N.I	34.65	647(+)	647 501 467 347 321 303 285	-
6	Viscidulin III –*O*– glucoside	35.22	507	345 330 315	-
7	Tetrahydroxyflavone	37.69	285	285 268 241 217 199 177 151 133 107	43.056 ± 0.112
8	Iridin	40.20	521	383 359 344 329 313 300 285 212	21.472 ± 0.415
9	Eriodictyol (4′-hydroxynaringenin)	42.25	289(+)	289 271 247 179 163 153 147	45.679 ± 0.782
10	Puerarin	43.24	415	415 295 267 253 223	7.003 ± 0.022
11	Viscidulin III	43.88	347(+)	347 332 317 314 289 286 183 169 150 142	-
12	Pentahydroxyflavone	45.11	301	301 283 269 241 225 197 179 165 161 139 133 124 107	20.622 ± 0.036
13	N.I	52.55	675	675 529 481 361 335 317 285	-
14	Baicalin	53.67	447(+)	447 343 271 253 225 169 149 123105	3.035 ± 0.013
15	Scutellarein	56.25	285	285 267 257 239 213 195 185 167 165 137 119 117	21.017 ± 0.133
16	Isoscutellarein	57.35	285	285 267 257 241 239 229 213 185 167 165 137 119 117	7.882 ± 0.009

^a^Quantity data are the mean ± SD of three replicates determinations by HPLC-UV method.

N.I: Not Identified.

### Effects of flavonoids on L6 cells viability

**Figure 2:**
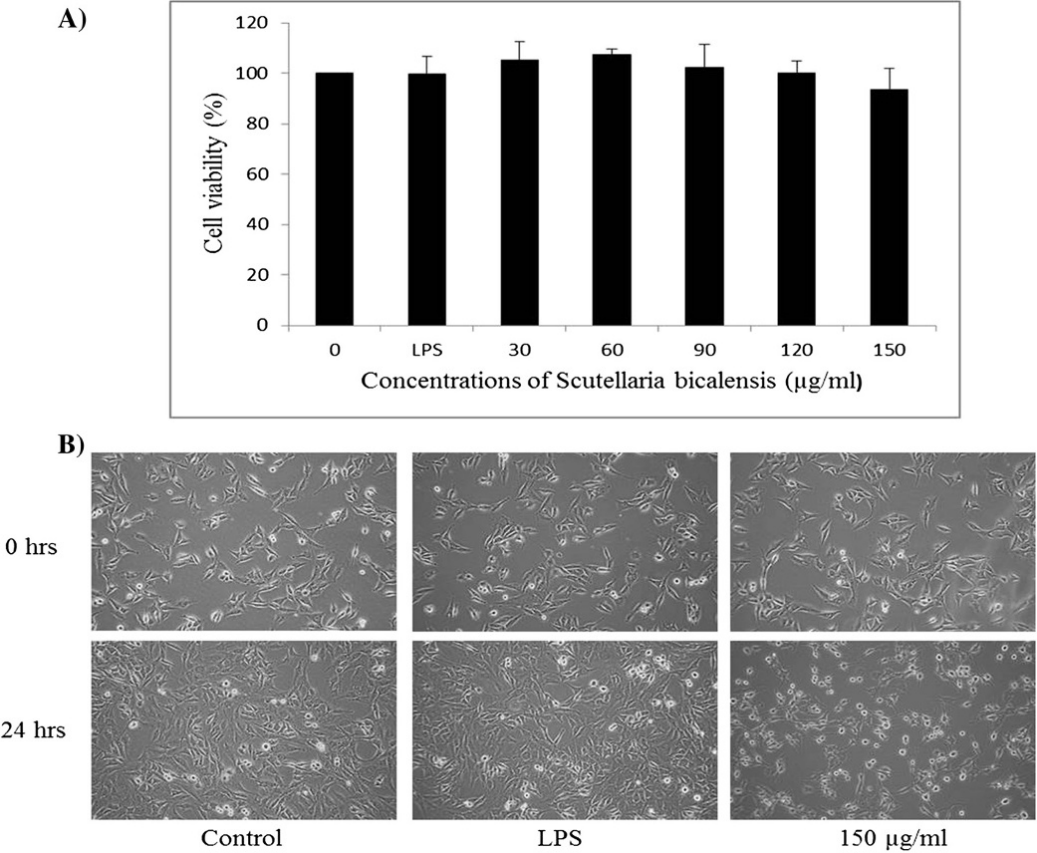
**The effect of the flavonoids on L6 cells viability.** MTT assay were conducted at various concentrations (0, 30, 60, 90, 120 and 150 *μ*g/mL) in the absence or presence of lipopolysaccharide (1 *μ*g/mL) **(A)**. The morphological changes L6 cells were visualized by optical microscopy (×400) **(B)**. Results are expressed as the mean ± SD of three independent experiments.

To determine the cytotoxic effects of the flavonoids on L6 cells, MTT assay was performed after treated with flavonoids at various concentrations (30, 60, 90, 120 and 150 *μ*g/mL) and incubated the cells for 24 h in the presence or absence of LPS (1 *μ*g/mL). The results showed that LPS (1 *μ*g/mL) and flavonoids of 30–150 *μ*g/mL has no cytotoxicity to L6 cells but cell viability was start to decrease at 150 *μ*g/mL (Figure 2A). Therefore, flavonoids concentrations up to 150 *μ*g/mL were used for subsequent experiments. The cell morphology of L6 cells after flavonoids treatment in the presence or absence of LPS (1 μg/mL) were monitored under optical microscopy (×400) after 24 h incubation (Figure 2B).

### 2-DE and MALDI-TOF/MS

**Figure 3:**
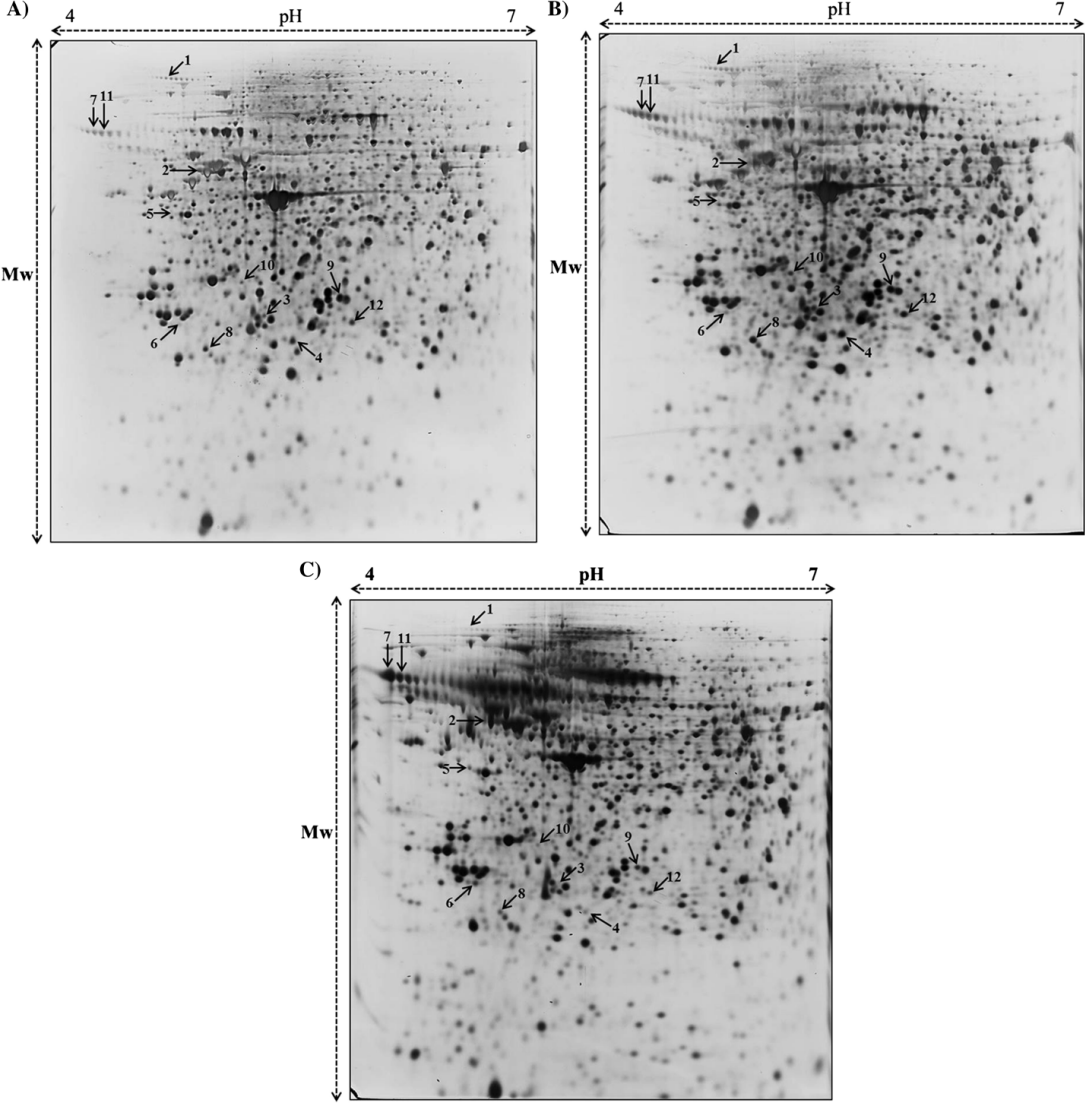
**Protein profiles of L6 cells of (A) the control, (B) LPS treated and (C).** 150 μg of flavonoid treated groups. Proteins were separated on pH 4–7, 17 cm linear IPG strips in the first dimension and 12% SDS-PAGE in the second dimension. The gels were silver stained.

The 150 μg of total proteins from the L6 cells of the control, LPS treated, and flavonoid treated groups were extracted and resolved by 18 cm, pH 4–7 IPG strips in the first dimension and 12% SDS-PAGE in the second dimension. Approximately 500 protein spots were detected in these silver-stained 2-DE maps. All protein spots exhibited molecular weights (MWs) of 10–175 kDa. The differences in the spot intensity were identified as quantitative changes. A total of 26 differentially expressed protein spots were identified (more than two-fold was considered significant, p < 0.05) (Figure 3), and 12 were successfully detected by MALDI-TOF/MS. The identified proteins name, accession number, number of matching peptides, theoretical isoelectric point (pI), molecular weights, sequence coverage and expression patterns in L6 cells were listed in Table 2. The majority of the proteins were involved in inflammatory responses such vimentin (Vim), T-box transcription factor TBX3 (Tbx3), annexin A1 (Anxa1), annexin A2 (Anxa2) and annexin A5 (Anxa5).

**Table 2:** **List of proteins identified from LPS stimulated L6 cells after flavonoids treatment by MALDI-TOF MS**

Spot no	Accession number ^1^	Protein name ^1^	Theoretical Mr (Da) ^1^	Theoretical pI value ^1^	Matched peptides	Sequence coverage	Mowse score ^2^
1	Q9QXU8	Cytoplasmic dynein 1 light intermediate chain 1	56985	6.13	8	16	61
2	P31000	Vimentin	53757	5.06	26	52	108
3	Q7TST9	T-box transcription factor TBX3	79684	7.72	8	12	51
4	P09812	Glycogen phosphorylase, muscle form	97725	6.91	20	25	58
5	Q29RW1	Myosin-4	223653	5.58	33	17	56
6	Q9JII3	Prolactin-2A1	26148	6.44	7	29	51
7	Q9JHZ4	GRIP1-associated protein 1	96300	5.17	10	13	54
8	Q8CGR3	Alpha-S2-casein-like B	20004	9.62	7	43	53
9	P02770	Serum albumin	70682	6.09	16	28	76
10	P14668	Annexin A5	35779	4.93	16	45	79
11	Q07936	Annexin A2	38939	7.55	14	49	62
12	P07150	Annexin A1	39147	6.97	12	42	57

^1^Accession number, protein name, theoretical molecular weight (Da) and pI from SWISS-PROT database identified by MALDI-TOF/MS.

^2^Score is -10*log (p), where p is the probability that the observed match is a random event, Protein scores greater than 51 are considered to be significant (p < 0.05).

### Effect of flavonoids on expression of COX-2, iNOS and Annexin A2 proteins in LPS-stimulated L6 skeletal muscle cells

**Figure 4:**
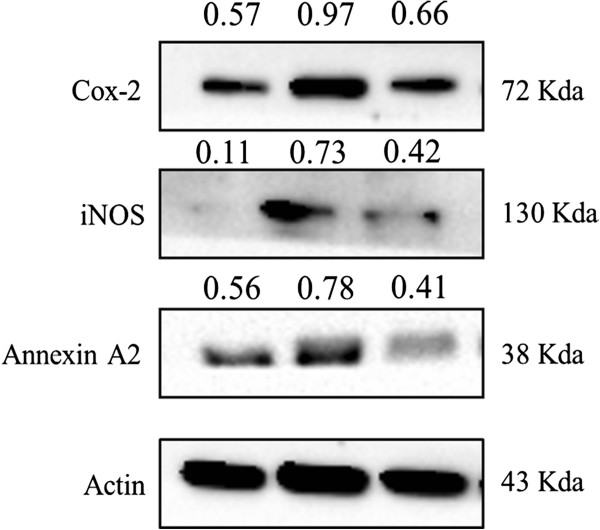
**Effects of flavonoids on lipopolysaccharide induced L6 skeletal muscle cells.** L6 cells were untreated or treated with the indicated concentration of LPS and flavonoids for 24 h, and COX-2, iNOS and Annexin A2 protein levels in the cell lysates were assayed by Western blot analysis. The experiments were conducted at least three replications.

**Figure 5:**
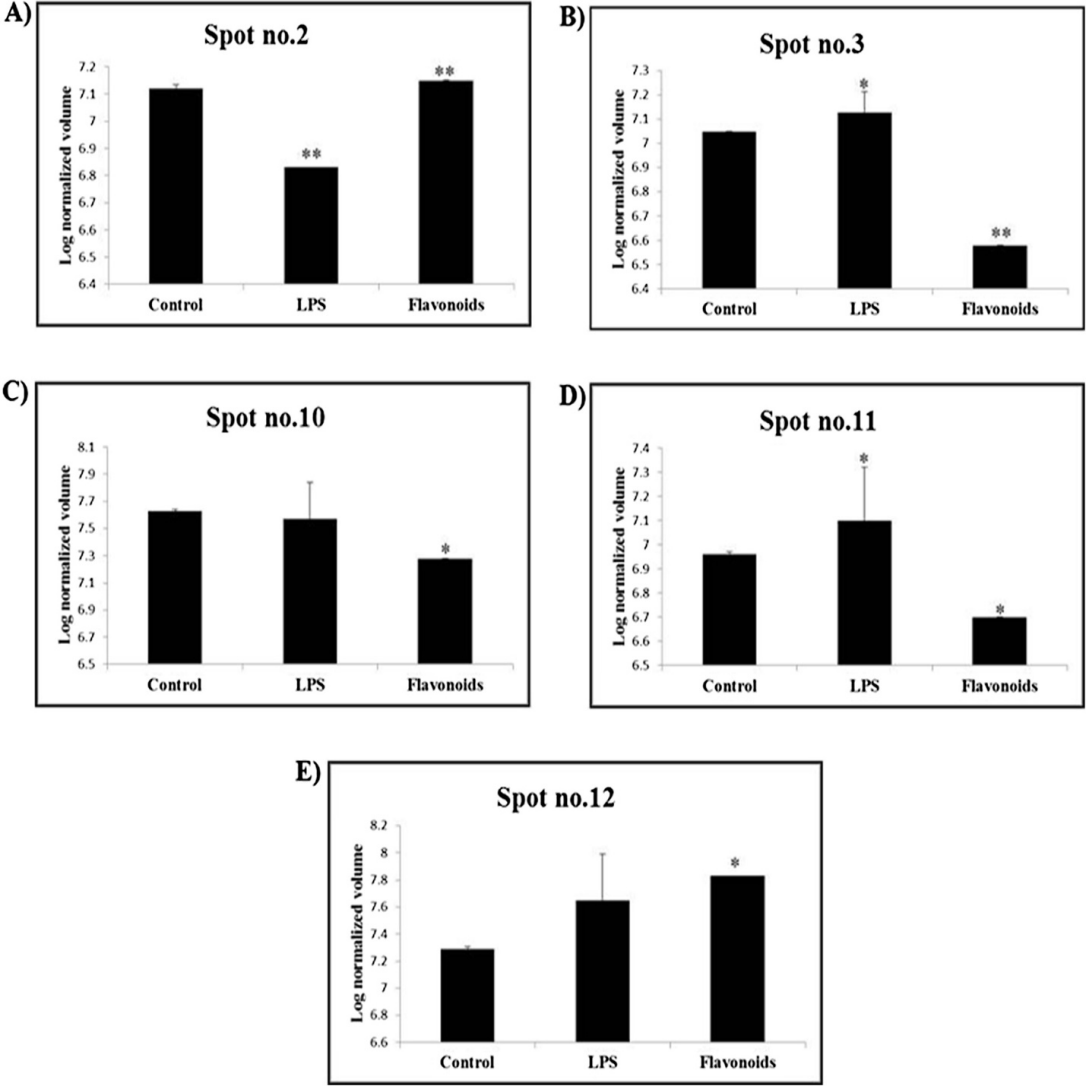
**Expression profiles.** Vimentin **(A)**, T-box transcription factor TBX3 **(B)**, Annexin A5 **(C)**, Annexin A2 **(D)** and Annexin A1 **(E)** from L6 cells 2-DE maps of control, LPS and flavonoids treated groups. Three independent experiments were performed and the mean ± SD was plotted (*P < 0.05, **P < 0.01 compared with control).

The effect of flavonoids on the expression of COX-2 and iNOS proteins were determined by western blot. In LPS-stimulated L6 skeletal muscle cells, the expression of COX-2 and iNOS proteins were remarkably increased and pretreatment with flavonoid (150 *μ*g/mL) attenuated protein expression (Figure 4). These results were suggest that flavonoids can inhibit LPS-induced inflammation by suppression COX-2 and iNOS expression at translational levels. Moreover, an anti-inflammatory protein, Anxa2 (unique annexins) expression was increased in LPS-treated cells and, inhibited by flavonoids in LPS stimulated L6 skeletal muscle cells (Figures 4 and 5D). These results consistent with 2-DE results that LPS can induce inflammation in L6 cells by increasing Anxa2 protein and, that can be inhibited by flavonoids which isolated from *S. baicalensis* G.

### GO analysis

**Figure 6:**
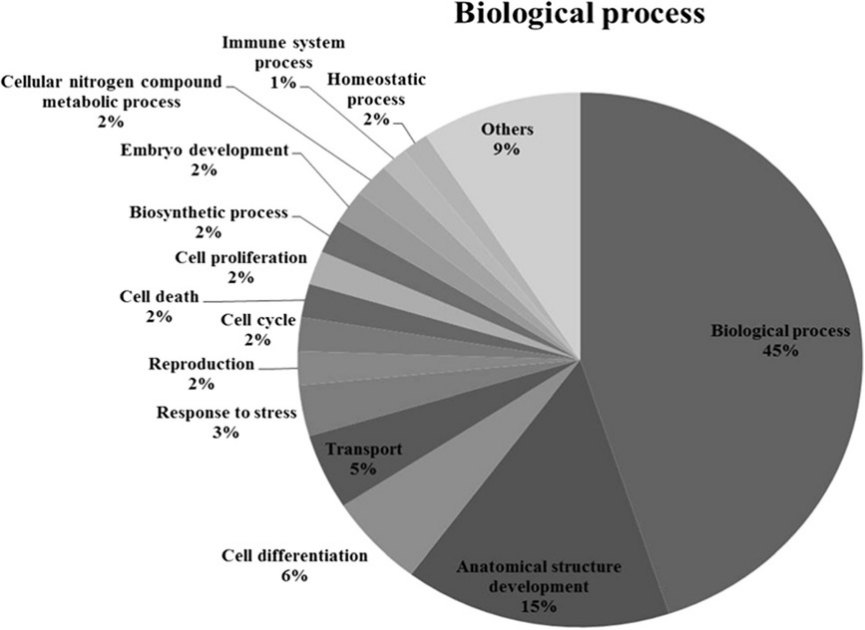
**Gene ontology analysis (GO).** GO analysis of the identified proteins in LPS stimulated L6 cells after treated with flavonoids. Pie charts representing the distribution of the identified proteins according to their biological function. This classification was produced based on an analysis using GOSlimViewer tool at Agbase (http://www.agbase.msstate.edu/).

The most notable functional categories in relation to the protein expression pattern are shown in Figure 6. The strongest associations were with biological processes (44%) (GO: 0008150). Another 15% of the associations were with anatomical structure development (GO: 0048856), whereas 5.5% were associated with cell differentiation (GO: 0030154) and 4.5% with transport (GO: 0006810).

## Discussion

*S. baicalensis* G. has been used as traditional herbal medicine in Asian countries because of its medicinal properties including anti-viral, anti-microbial, anti-inflammatory and anti-cancer properties [22, 23]. In the current study, the anti-inflammatory effects of flavonoids isolated from *S. baicalensis* G. in LPS stimulated L6 skeletal muscle cells using proteome techniques. The western blot results shows that the expression of COX-2 and iNOS proteins were remarkably increased by LPS and pretreatment with flavonoid (150 *μ*g/mL) attenuated protein expression in LPS-stimulated L6 skeletal muscle cells. These data suggests that flavonoids can inhibit inflammation by suppression of LPS-induced COX-2 and iNOS expression. Totally, 12 differentially expressed proteins were identified in L6 skeletal muscle cells after treated with LPS alone and combined with flavonoids using 2-DE coupled with MALDI-TOF/MS analysis (Figure 3 and Table 2). Proteomic analysis revealed that identified proteins were the involved in inflammatory responses due to flavonoids from *S. baicalensis* G. effects.

Firstly, vimentin expression is decreased by LPS and increased by flavonoids in L6 skeletal muscle cells (Figure 5A). It has been reported that vimentin is secreted by activated human macrophages and that’s involved in immune functions of this cell type, as extracellular vimentin was found to be involved in bacterial killing and the generation of oxidative metabolites [24]. Schietke et al. [25] have demonstrated that mutations in vimentin were found to disrupt the cytoskeleton in fibroblasts and delay execution of apoptosis [25]. Another important protein, T-box transcription factor TBX3, is increased in LPS treated groups and decreased in flavonoids treated groups (Figure 5B). Previous studies have reported that T-box genes/proteins such as TBX2 and TBX3 are overexpressed in several neoplasms [26–28]. Also, Peres and prince [29] have demonstrated that increased levels of TBX3 are sufficient to promote tumor formation and invasion of non-tumorigenic melanoma cells in vivo [29]. These results suggest that flavonoids isolated from *S. baicalensis* G. protected the LPS induced inflammation in L6 cells.

The annexins are a family of phospholipid-binding proteins [30], and functionalize in the processes of endo- and exocytosis, anti-inflammation, anticoagulation, signal transduction, ion channel formation, cell proliferation, division and apoptosis, tumor development, invasion, metastasis and drug resistance [31, 32]. In the current study, altered expression in L6 cells of Anxa1, Anxa2 and Anxa5 found in LPS and flavonoids treated groups compared to control. Annexin A1 (Anxa1), also known as lipocortin-1, an anti-inflammatory factor, originally reported as glucocorticoid-induced protein with anti-phospholipase activity that has been shown to regulate diverse cellular functions such as hormones secretion, vesiculation, inflammatory response, apoptosis and differentiation [33]. Also, Anxa1 exhibits profound inhibitory actions on leukocyte transmigration and activation, which leading to the resolution of inflammation [34]. Even though, Anxa1 is an anti-inflammatory protein, its significantly increased in flavonoids treated L6 cells expression, meanwhile no significant changes found in LPS treated groups (Figure 5E). Interestingly, previous study has reported that Anxa1 phosphorylation may have a role in the anti-inflammatory effects in response to drugs [35]. These results indicates that flavonoids from *S. baicalensis* G have anti-inflammatory properties on LPS stimulated L6 cells by increasing the Anxa1 expression. However, further studies need to be proved the relationship between the flavonoids effects and Anxa1 phosphorylation.

It has been reported that Anxa5 up-regulation correlates with the inflammation-associated carcinogenesis of fibrosarcoma [36]. Also, Anxa5 might bind to phospholipid with high affinity on apoptotic cell membrane and it signals to immunity system [37]. In our study, Anxa5 expression was significantly decreased in flavonoids treated groups and no changes found in LPS treated groups (Figure 5C). In addition, Anxa2 is a unique among annexins and also an anti-inflammatory protein which involved in NF-kB signaling pathway [38]. The increased expression of Anxa2 was found in LPS treated groups and significantly decreased exprssions were observed in flavonoids treated groups (Figure 5D). These data suggest that LPS can induce inflammation in L6 cells by increasing Anxa5 and Anxa2 protein and, that can be inhibited by flavonoids which isolated from *S. baicalensis* G.

## Conclusions

In conclusion, we demonstrated that the anti-inflammatory effects of flavonoids from *S. baicalensis* G. on LPS stimulated L6 cells using proteomic approach. Flavonoids inhibited the expression of COX-2 and iNOS proteins in LPS-stimulated L6 skeletal muscle cells. Also, the 14 differentially expressed proteins were successfully identified by MALDI-TOF/MS that’s were involved in inflammatory responses such vimentin, T-box transcription factor TBX3, annexin A1, annexin A2 and annexin A5. Further, the identified proteins were involved in inflammatory responses and, that Annexin A2 was validated by immune-blotting. Moreover, our study is the first proteome study provide the anti-inflammatory mechanism of flavonoids from *S. baicalensis* G. in LPS stimulated L6 cells. These research findings would be helpful to study further the muscle cell inflammatory mechanisms.
